# Anaplastic Multiple Myeloma With a Novel Proliferation of Pleomorphic Binuclear Plasma Cells

**DOI:** 10.7759/cureus.63597

**Published:** 2024-07-01

**Authors:** Mark T Cunningham, Daniel Farrell, Sayedamin Mostofizadeh, Saurav Chopra

**Affiliations:** 1 Pathology and Laboratory Medicine, University of Kansas Medical Center, Kansas City, USA

**Keywords:** binuclear, pleomorphic, nuclei, morphometry, morphometric, morphology, pleural fluid, plasma cell, anaplastic, multiple myeloma

## Abstract

This study describes an unusual case of multiple myeloma that progressed to anaplastic multiple myeloma in the pleural fluid. The Wright-stained cytospin of the pleural fluid showed a predominant population of mononuclear plasma cells with pleomorphic nuclei, characterized by both small and large nuclei, which is typical of anaplastic multiple myeloma. However, there were also more binuclear plasma cells with pleomorphic nuclei. Morphometric analysis showed that the mean nuclear length was 1.9-fold and 2.3-fold higher in the large nuclei compared to the small nuclei for the mononuclear plasma cells and binuclear plasma cells, respectively (p<0.001). The patient received B cell maturation antigen chimeric antigen receptor T cell (CAR-T) therapy for relapsed disease, with a significant reduction of the serum monoclonal paraprotein level at day 51 post-therapy. Pathologists should be aware that pleomorphic binuclear plasma cells can be part of the morphologic spectrum in anaplastic multiple myeloma.

## Introduction

Multiple myeloma is a neoplastic proliferation of plasma cells that usually originates in the bone marrow [1]. Extramedullary involvement is a feature of advanced disease and can rarely involve the pleural fluid [2]. Neoplastic plasma cells can vary in their morphology, ranging from mature forms indistinguishable from normal plasma cells to plasmablastic, lymphoplasmacytic, pleomorphic, and other morphologic subtypes [1]. Anaplastic multiple myeloma is a rare morphologic variant characterized by pleomorphic plasma cells with large nuclei, as first reported in 1983 by Foucar et al. [3].

We report a rare case of multiple myeloma that transformed into anaplastic multiple myeloma in the pleural fluid. Our case was unique in that the Wright-stained cytospin of the pleural fluid showed pleomorphic binuclear plasma cells. This study describes the clinical, pathologic, and morphometric features of this unusual manifestation of anaplastic multiple myeloma.

## Case presentation

A 73-year-old female presented with a chief complaint of dyspnea and fatigue for two days. Past medical history was significant for IgG kappa multiple myeloma, diagnosed seven years earlier as a stage one disease. Previous myeloma therapy included induction with RVD (lenalidomide, bortezomib, dexamethasone) at diagnosis followed by lenalidomide maintenance, RVD for first bone marrow relapse, DPD (daratumumab, pomalidomide, dexamethasone) for second bone marrow relapse, KPD (carfilzomib, pomalidomide, dexamethasone) for third bone marrow relapse, and KCD (carfilzomib, cyclophosphamide, dexamethasone) for fourth bone marrow relapse and bridging therapy prior to planned chimeric antigen receptor T cell (CAR-T) therapy. The patient declined autologous hematopoietic stem cell transplantation early after diagnosis. A routine bone marrow biopsy was performed 60 days prior to the current presentation, and the morphology, flow cytometry, conventional cytogenetics, and fluorescence in situ hybridization results are shown in Table 1.

**Table 1:** Bone marrow biopsy results 60 days prior to presentation with pleural effusion

Laboratory parameter	Result	Reference range
Bone marrow morphology	Hypercellular marrow (50-60%), normal myelopoiesis, normal erythropoiesis, decreased megakaryopoiesis, and 48% atypical plasma cells. Plasma cell subtypes: 90% small mononuclear, 6% large mononuclear, and 4% small binuclear. Multinucleated plasma cells with three or more nuclei were not seen. Plasma cell morphology: round to oval nuclear shape, peripherally located nucleus, smooth nuclear contour, dispersed chromatin with multifocal small chromatin condensations, prominent single nucleolus with smooth contour, predominantly absent perinuclear hof, abundant basophilic cytoplasm, absent vacuoles	Normocellular marrow (30-40%), normal trilineage hematopoiesis, 0-1% plasma cells
Flow cytometry	Monoclonal kappa restricted plasma cells (47%) with increased expression of CD56, and decreased expression of CD19, CD27, CD45, and CD81	0-1% polyclonal plasma cells
Conventional cytogenetic karyotype	53,X,-X, add(1)(p13),t(2;10)(q21;p11.2), +5,+7,+9,+9,del(11)(p15p13),+add(15)(p11.2),+19,-20,+21,-22,+r,+2mar(13)/46,XX(7)	46,XX (20 metaphases)
Fluorescence in situ hybridization for gain of 1p1q (3 copies)	Present in 77.0% of interphase nuclei; standard nomenclature: nuc ish(CDKN2Cx1,CKS1Bx3)(77/100); loss of 1p, gain of 1q	Present in <3.0% of interphase nuclei
Fluorescence in situ hybridization for amplification of 1p1q (>4 copies)	Present in 17.0% of interphase nuclei; standard nomenclature: nuc ish(CDKN2Cx2,CKS1B amp)(17/100) amplification of 1q	Present in <3.0% of interphase nuclei
Fluorescence in situ hybridization for t(11;14)	Present in 0% of interphase nuclei; standard nomenclature: nuc ish(CCND1/MYEOVx3,IGHx2)(73/100) Extra CCND1/MYEOV signal	Present in <3.0% of interphase nuclei
Fluorescence in situ hybridization for deletion of TP53	Present in 0% of interphase nuclei; standard nomenclature: nuc ish(TP53,D17Z1)x2(100)	Present in <6.0% of interphase nuclei

The physical exam was unremarkable. A chest radiograph showed mild bilateral pleural effusions. Thoracentesis was performed. Laboratory studies are summarized in Table 2.

**Table 2:** Pertinent laboratory results

Laboratory parameter	Result	Reference range
Hemoglobin	8.6 g/dL	12.0-15.0 g/dL
Platelets	147,000 /uL	150,000-400,000 /uL
White blood cell count	1,500 /uL	4,500-11,000 /uL
Neutrophil count	1,400 /uL	1,800-7,000 /uL
Lymphocyte count	0 /uL	1,000-4,800 /uL
Monocyte count	100 /uL	0-800 /uL
Lactate dehydrogenase	197 IU/L	100-190 IU/L
Creatinine	0.81 mg/dL	0.40-1.00 mg/dL
Uric acid	4.3 mg/dL	2.0-6.0 mg/dL
Potassium	3.8 mmol/L	3.5-5.1 mmol/L
Calcium	8.0 mg/dL	9.0-11.0 mg/dL
Corrected calcium	9.3 mg/dL	9.0-11.0 mg/dL
Albumin	2.4 g/dL	3.5-5.0 g/dL
Total protein	10.2 g/dL	6.0-8.0 g/dL
Serum immunofixation electrophoresis	IgG kappa paraprotein	Absent paraprotein
Serum paraprotein	5.58 g/dL	0 g/dL
Pleural fluid, red blood cells	13,065 /uL	0 /uL
Pleural fluid, white blood cells	3,907 /uL	0 /uL
Pleural fluid, monocyte count	2%	0%
Pleural fluid, plasma cell count	98%	0%
Pleural fluid, lactate dehydrogenase	177 IU/L	67-140 IU/L
Pleural fluid, total protein	6.5 g/dL	<1.1 g/dL
Pleural fluid, flow cytometry	96% monoclonal kappa plasma cells with increased expression of CD56 and CD117	Polyclonal plasma cells

**Figure 1:**
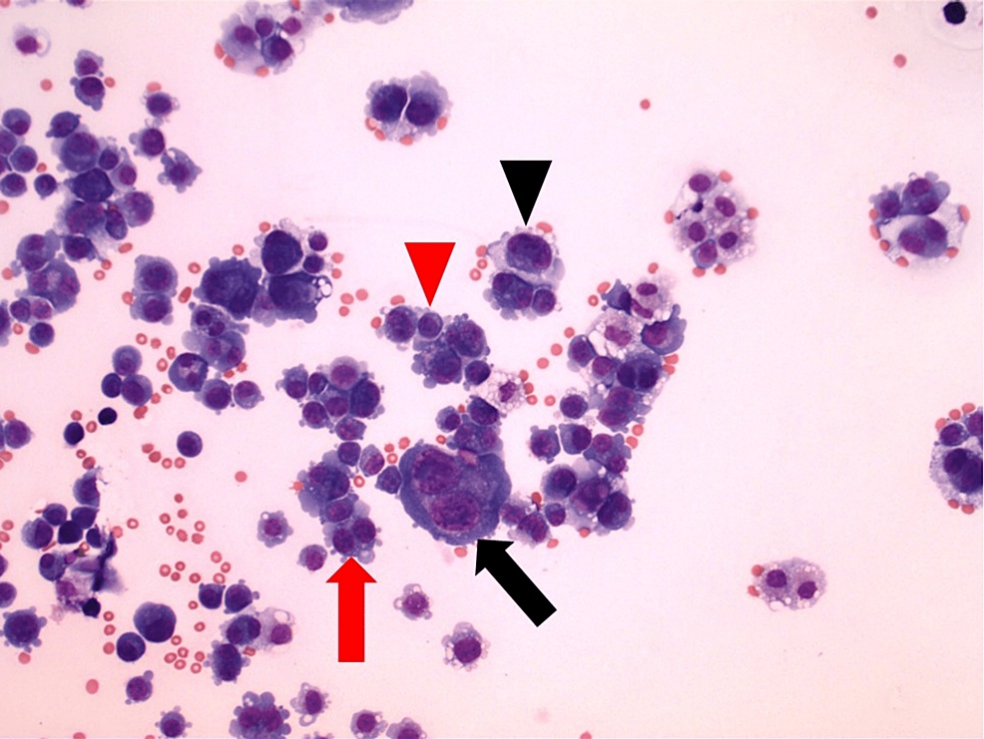
Pleural fluid cytospin showing atypical plasma cells (Wright stain, 200x) Mononuclear plasma cell with small nucleus (red arrowhead), mononuclear plasma cell with large nucleus (black arrowhead), binuclear plasma cell with small nuclei (red arrow), and binuclear plasma cell with large nuclei (black arrow)

The Wright-stained cytospin of the pleural fluid showed 98% atypical plasma cells with pleomorphic morphology. There were five distinct plasma cell subtypes based on the number of nuclei per cell and the size of the nuclei. These subtypes included mononuclear plasma cells with small nuclei, mononuclear plasma cells with large nuclei, binuclear plasma cells with small nuclei, binuclear plasma cells with large nuclei, and multinucleated plasma cells (three to five nuclei per cell) with small nuclei. A low-power view of the atypical plasma cells in the pleural fluid is shown in Figure 1.

**Figure 2:**
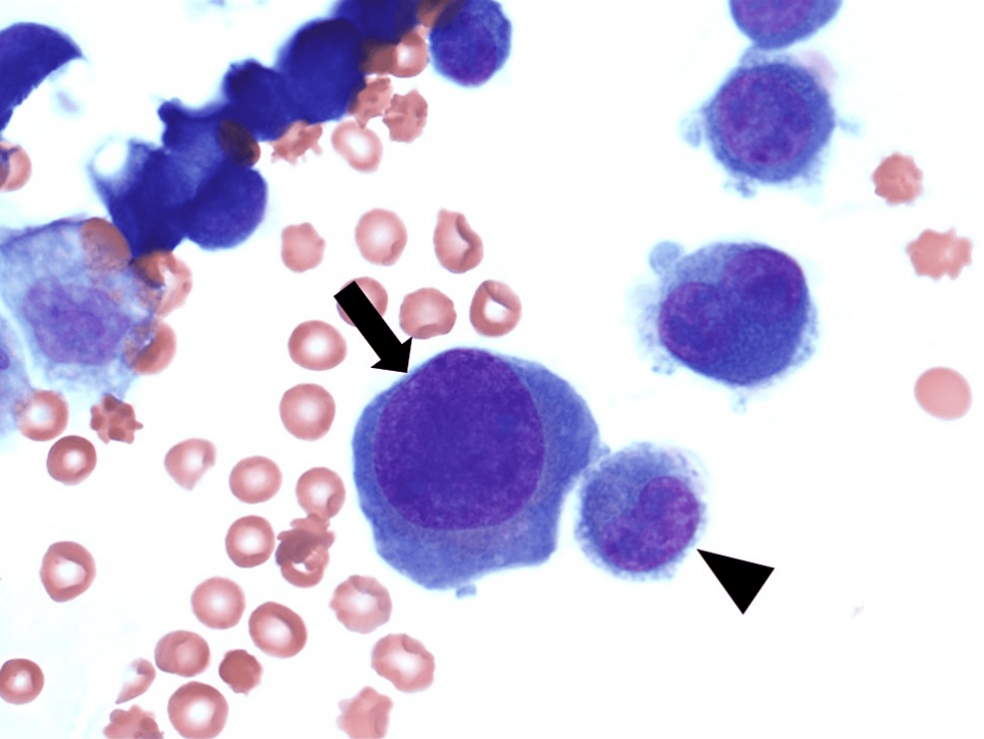
Pleural fluid cytospin showing a mononuclear plasma cell with a large nucleus (Wright stain, 1000x) Mononuclear plasma cell with large nucleus (arrow) and mononuclear plasma cell with small nucleus (arrowhead)

A typical example of a mononuclear plasma cell with a large nucleus is shown in Figure 2.

**Figure 3:**
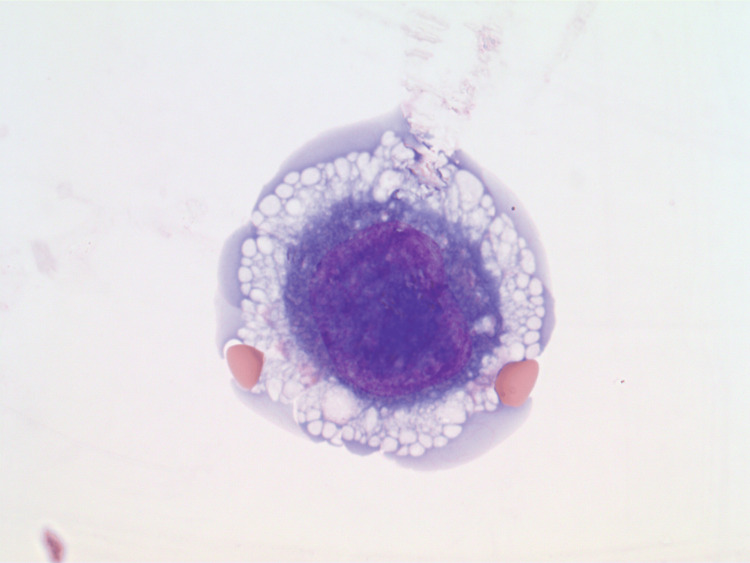
Pleural fluid cytospin showing a mononuclear plasma cell with a large nucleus and abundant giant cytoplasmic vacuoles (Wright stain, 1000x)

A typical example of a mononuclear plasma cell with a large nucleus and abundant giant cytoplasmic vacuoles is shown in Figure 3.

**Figure 4:**
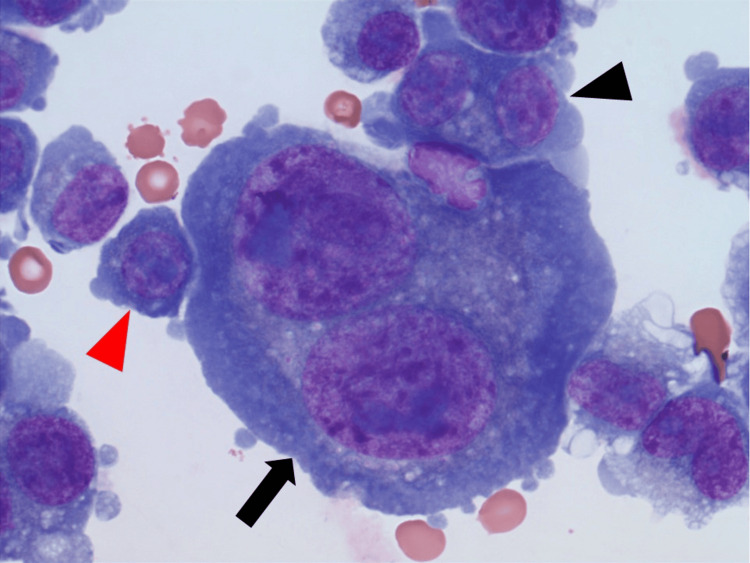
Pleural fluid cytospin showing a binuclear plasma cell with two large nuclei (Wright stain, 1000x) Binuclear plasma cell with large nuclei (black arrow), binuclear plasma cell with small nuclei (black arrowhead), and mononuclear plasma cell with small nucleus (red arrowhead)

A typical example of a binuclear plasma cell with large nuclei is shown in Figure 4.

**Figure 5:**
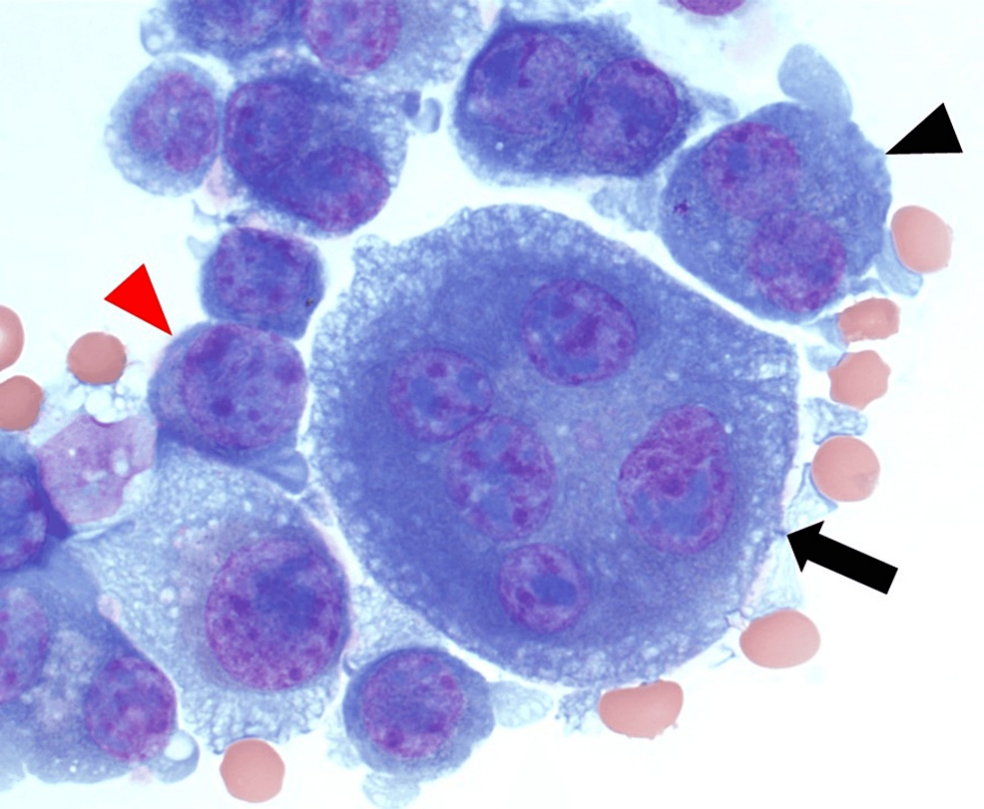
Pleural fluid cytospin showing a multinucleated plasma cell with five small nuclei (Wright stain, 1000x) Multinucleated plasma cell with five small nuclei (black arrow), binuclear plasma cell with small nuclei (black arrowhead), and mononuclear plasma cell with small nucleus (red arrowhead)

A typical example of a multinucleated plasma cell with small nuclei is shown in Figure 5.

Morphometric analysis of the different plasma cell subtypes was performed on digital microscopic images of the Wright-stained cytospin at 1000x magnification using an Olympus BX41 microscope (Evident Scientific, Inc., Waltham, MA), an Infinity 1 digital camera (Lumenara Corporation, Ottawa, ON, Canada), and Infinity Analyze software release 6.5.3 (Lumenera Corporation). Nuclear length was defined as the length of the nucleus at its maximum dimension. The mean nuclear length was 1.9-fold higher and 2.3-fold higher in the large nuclei compared to the small nuclei in the mononuclear and binuclear plasma cells, respectively. The mean nuclear length of the nuclei within the multinucleated plasma cells was similar to the mean nuclear length of the small nuclei within the mononuclear and binuclear plasma cells. The percentages, nuclear lengths, and morphologic descriptions of the different plasma cell subtypes are shown in Table 3.

**Table 3:** Analysis of plasma cell subtypes in pleural fluid NA: not applicable ^1 ^Control specimen is a pleural fluid cytospin from a patient with reactive plasmacytosis. ^2 ^Nuclear length is the mean +/- 1 standard deviation. ^3 ^All p-values are based on the two-tailed t-test. ^4 ^p-value is a comparison with the control small nuclei. ^5 ^p-value is a comparison with the case report mononuclear small nuclei. ^6 ^p-value is a comparison with the case report binuclear small nuclei.

Specimen	Plasma cell subtype	Percentage	Nuclear length (um)^2^	P-value^3^	Morphologic description
Control^1^	Mononuclear, small nuclei	NA	20.09 +/- 2.38 (n=20 nuclei)	NA	NA
Case report	Mononuclear, small nuclei	55%	23.39 +/- 2.77 (n=29 nuclei)	<0.001^4^	Round to oval nuclear shape, smooth nuclear contour, dispersed chromatin with multifocal small chromatin condensations, single prominent nucleolus with smooth contour, abundant basophilic cytoplasm, minimal vacuolation
	Mononuclear, large nuclei	24%	44.51 +/- 8.36 (n=8 nuclei)	<0.001^5^	Round to oval nuclear shape, smooth nuclear contour, dispersed chromatin with multifocal large chromatin condensations, multiple giant nucleoli with irregular contour, abundant basophilic cytoplasm, vacuolation
	Binuclear, small nuclei	15%	20.65 +/- 1.90 (n=24 nuclei)	0.39^4^	Round to oval nuclear shape, smooth nuclear contour, dispersed chromatin with multifocal small chromatin condensations, single to multiple prominent nucleoli with smooth contour, abundant basophilic cytoplasm, minimal vacuolation
	Binuclear, large nuclei	5%	47.67 +/- 4.20 (n=8 nuclei)	<0.001^6^	Round to oval nuclear shape, smooth nuclear contour, dispersed chromatin with multifocal large chromatin condensations, multiple giant nucleoli with irregular contour, abundant basophilic cytoplasm, vacuolation
	Multinucleated, small nuclei	1%	23.57 +/- 4.22 (n=16 nuclei)	0.007^4^	Round nuclear shape, smooth nuclear contour, dispersed chromatin with multifocal large chromatin condensations, multiple giant nucleoli with irregular contour, abundant basophilic cytoplasm, vacuolation

The patient received a B cell maturation antigen CAR-T cell infusion using ciltacabtagene autoleucel (cilta-cel). Therapy was well tolerated, and at day 51 post-therapy, there was an 89% reduction in the serum IgG kappa paraprotein level (from 5.58 g/dL to 0.59 g/dL).

## Discussion

To our knowledge, this is the first reported case of anaplastic multiple myeloma with pleomorphic binuclear plasma cells [1]. These cells were identified morphologically on the Wright-stained cytospin of the pleural fluid. Morphometric analysis demonstrated two populations of binuclear plasma cells, one with small nuclei and the other with large nuclei having more than double the nuclear length of the small nuclei. There was also a predominant population of mononuclear plasma cells with small nuclei and large nuclei, as well as a minor population of multinucleated plasma cells with small nuclei.

Different morphologic subtypes of plasma cells have been described in anaplastic multiple myeloma. These include the large mononuclear plasma cell [3-4], immunoblastic plasma cell [5], multilobated plasma cell [6-7], cleaved plasma cell [7], spindled plasma cell [8], and multinucleated plasma cell [3,9-10]. Our case demonstrated that the large binuclear plasma cell is an additional plasma cell subtype in anaplastic multiple myeloma.

Pleural fluid metastasis is a rare extramedullary manifestation that affects 0.8% of multiple myeloma patients [2]. It can occur at presentation or at relapse and is sometimes accompanied by bone marrow involvement. The prognosis is poor, with a median survival of four months after the onset of pleural fluid metastasis [11]. Our case is the first report of anaplastic multiple myeloma involving the pleural fluid.

There are multiple cytogenetic abnormalities that have been reported in anaplastic multiple myeloma [12-13]. These include amplification of 1q21 (CKS1B), del(13q14.3), del(17p), t(4;14), complex karyotype, and extreme hyperploidy. There is a high prevalence of CKS1B amplification, del(17p), and t(4;14) in anaplastic multiple myeloma compared to non-anaplastic cases [13]. The gain of chromosome 1q21 is associated with a poor prognosis [14]. Our case had a hyperploid karyotype with multiple numeric and structural abnormalities, including both gain and amplification of chromosome 1q21 (CKS1B), consistent with previously reported cases.

Anaplastic multiple myeloma has a poor prognosis. In 1990, the mean survival was 3.2 months after the onset of anaplastic disease [5]. In 2022, the median survival was 1.5 years, compared to 4.9 years for non-anaplastic multiple myeloma [15]. We found no previous cases in the literature describing the therapeutic response of this myeloma variant to CAR-T cell therapy. To our knowledge, this is the first report of anaplastic multiple myeloma showing a favorable early response to B cell maturation antigen CAR-T cell therapy.

## Conclusions

This case report demonstrated that anaplastic multiple myeloma can show pleomorphic binuclear plasma cell morphology. Morphometric analysis was useful in characterizing the size of individual nuclei within the various plasma cell populations. In addition, anaplastic multiple myeloma can present as a pleural effusion and can show a favorable early response to B cell maturation antigen CAR-T cell therapy. Pathologists should be aware that pleomorphic binuclear plasma cells can be part of the spectrum of morphologic abnormalities in anaplastic multiple myeloma.
